# Photoprotective Substances Derived from Marine Algae

**DOI:** 10.3390/md16110399

**Published:** 2018-10-23

**Authors:** Ratih Pangestuti, Evi Amelia Siahaan, Se-Kwon Kim

**Affiliations:** 1 Research Center for Oceanography, Indonesian Institute of Sciences (LIPI), Jakarta 14430, Indonesia; pangestuti.ratih@gmail.com; 2 Research and Development Division of Marine Bio-Industry, Indonesian Institute of Sciences (LIPI), West Nusa Tenggara 83552, Indonesia; eviamelia.siahaan@gmail.com; 3 Department of Marine Life Science, College of Ocean Science and Technology, Korea Maritime and Ocean University, Busan 606-791, Korea

**Keywords:** natural, bioactive, marine algae, photoprotective, substances

## Abstract

Marine algae have received great attention as natural photoprotective agents due to their unique and exclusive bioactive substances which have been acquired as an adaptation to the extreme marine environment combine with a range of physical parameters. These photoprotective substances include mycosporine-like amino acids (MAAs), sulfated polysaccharides, carotenoids, and polyphenols. Marine algal photoprotective substances exhibit a wide range of biological activities such as ultraviolet (UV) absorbing, antioxidant, matrix-metalloproteinase inhibitors, anti-aging, and immunomodulatory activities. Hence, such unique bioactive substances derived from marine algae have been regarded as having potential for use in skin care, cosmetics, and pharmaceutical products. In this context, this contribution aims at revealing bioactive substances found in marine algae, outlines their photoprotective potential, and provides an overview of developments of blue biotechnology to obtain photoprotective substances and their prospective applications.

## 1. Introduction

The ocean covers more than 70% of the Earth’s surface and represents an enormous resource of biodiversity. Marine organisms have adapted excellently to extreme environmental conditions with a range of physical parameters, such as pH, high salt concentration, low or high temperature, high-pressure, low nutrient availability, and low or high sun exposure [1]. The wide diversity in the biochemical composition of marine organisms provides an excellent reservoir to explore functional materials, many of which are rare or absent in other taxonomic groups. Large numbers of studies have demonstrated health-benefit effects of marine-derived functional materials [2,3]. 

**Figure 1:**
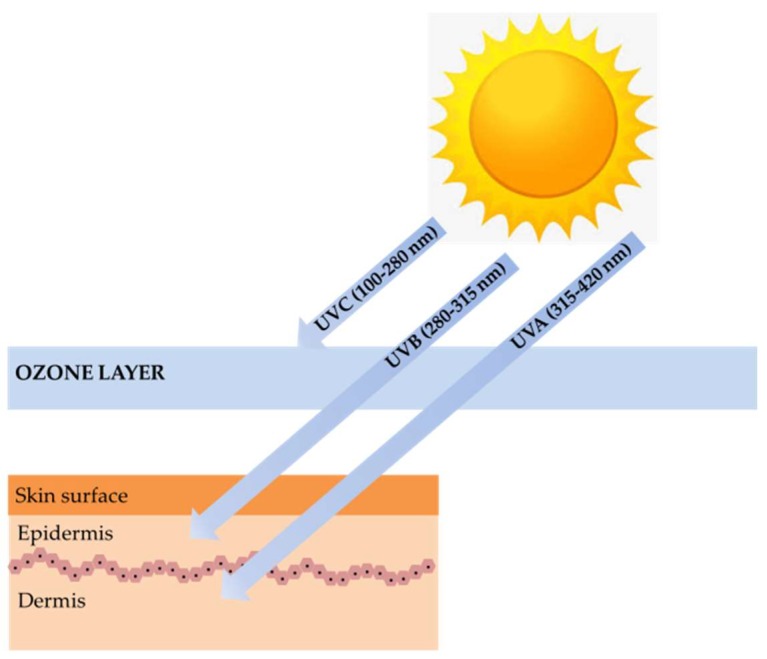
The ultraviolet (UV)-induced photodamage.

Marine algae are one of the most extensively studied marine organisms. These marine organisms have attracted special interest because they are good sources of nutrients and functional materials. Many studies have reported biological activities, including antioxidant, anti-cancer, anti-hypertension, hepatoprotective, immunomodulatory, and neuroprotective activity. Marine algae are already used in a wide range of foods, supplements, pharmaceuticals, and cosmetics and are often claimed to have beneficial effects on human health. One particular interesting feature in marine algae is their richness in photoprotective substances. Marine algae found in intertidal shores to a depth of 150 m are highly exposed to ultraviolet (UV) radiation. Therefore, to counteract and minimize photodamage induced by high UV radiation, photoprotective substances such as mycosporine-like amino acids (MAAs), sulfated polysaccharides, carotenoids, and polyphenols were synthesized [4]. These substances can be used for photoprotection to provide the skin with adequate protection against ultraviolet B (UVB) and ultraviolet A (UVA)-induced photodamage (Figure 1) [5].

Present approaches on the isolation and recovery of photoprotective substances from marine algae have been rapidly developing. Not only limited to organic solvent extraction, novel environmental friendly extraction and separation techniques, such as enzyme-assisted extraction (EAE), ultrasound assisted extraction (UAE), microwave assisted extraction (MAE), supercritical carbon dioxide (SC–CO_2_) and subcritical water extraction (SWE), have recently been applied to the development of photoprotective substances derived from marine algae. The recovery yield of photoprotective substances from marine algae depends on the technology applied and the marine algae species. In addition, the isolation process applied also affects photoprotective activity. Hence, this contribution focuses on photoprotective substances reported in marine algae. The most relevant studies on the photoprotective substances found in marine algae as well as their biological roles and photoprotective activity are discussed. Additionally, an overview of the developments of blue biotechnology and potential applications is also provided.

## 2. Photoprotective Substances Derived from Marine Algae

### 2.1. Sulfated Polysaccharides

Marine algae are considered as the most important source of non-animal sulfated polysaccharides, and chemical structures of these polymers differ according to class and species of algae [6,7]. Carrageenan and fucoidan are the major sulfated polysaccharides found in red and brown algae, respectively. Carrageenans are widely used in food, pharmacy, dairy, and cosmetic products due to the unique physical functional properties, such as thickening, gelling, emulsifying, and stabilizing properties [8]. These sulfated polysaccharides have been considered as safe additives for many commercial products in many countries. In addition to their unique physical functions, carrageenan composition in cosmetic and skin care products has often been found with antioxidant, tonifying, cleaning, hydrating, and revitalizing bioactivities. Recently, photoprotective effects of carrageenan (kappa, iota and lambda) in UVB-induced human keratinocytes (HaCaT) cells have been reported [9]. Carrageenan has shown significant protection against the detrimental effects of UVB-induced apoptosis in HaCaT cells and has decreased the release of reactive oxygen species (ROS). The accumulation of excess ROS has been related to skin diseases including skin aging and cancers. Therefore, antioxidants are usually viewed as preventive agents against UV-related skin diseases. We assumed that the photoprotective activity of carrageenan may also correlate to their immunomodulatory properties. Carrageenan has been known as an immunomodulator, which induces the expression of cyclooxygenase-2 (COX-2) and the release of prostaglandin-E_2_ (PGE_2_) [10]. Based on an in vivo experiment in SKH-1 hairless mice, Tripp et al. (2003) suggested that COX-2 expression is an important factor for keratinocyte survival and proliferation after acute UV irradiation. Inhibition of COX-2 expression has been demonstrated to reduce epidermal keratinocytes proliferation [11]. Taken together, it may be hypothesized that immunomulatory activities and ROS scavenging activities of carrageenan might play an important role in their photoprotective mechanisms. The addition of carrageenan to a broad spectrum of skin care and cosmetic products might decrease UV-induced photodamage compared with sunscreen alone. 

Fucoidan is the most commonly sulfated polysaccharide isolated from brown algae. In general, these linier polysaccharides have a backbone of α-linked l-fucose residues with various substitutions. Fucoidan structures and bioactivities are different among brown algae species [12]. Recent findings have reported the photoprotective activity of fucoidan isolated from brown algae including *Ecklonia cava*, *Undaria pinnatifida*, *Costaria costata*, and *Fucus evanescens* [13,14,15,16,17,18,19]. The photoprotective activity of fucoidan has been determined in UVB-irradiated human dermal fibroblast and mice models. Most studies report that the photoprotective activity of fucoidan is mediated through the suppression of matrix metalloproteinase-1 (MMP-1) activity. MMP-1 is a major enzyme implicated in the collagen damage and photoaging of UV-irradiated human skin. More precisely, these sulfated polysaccharides downregulate the expressions of NF-κB, which, in turn, diminish MMP-1 expression. Recently, it was reported that topical applications of low-molecular-weight fucoidan have stronger photoprotective activity than high-molecular-weight fucoidan [14]. The rationale for this is that low-molecular-weight fucoidan is mostly absorbed before irradiation. This low-molecular-weight fucoidan seems to be involved in photoprotective effects rather than UV filtering effects. 

Photoprotective activity in orally administered fucoidan, in addition to topical applications, has been reported. This information on the bioavailability of fucoidan might have stimulated further research on the relationship between the oral administration of fucoidan and their bioavailability, mode of action, and potency in skin care and cosmetic products. 

### 2.2. Carotenoids

Carotenoids are natural pigments found in all photosynthetic organisms (including plants, algae, and cyanobacteria) and some non-photosynthetic archaea, bacteria, fungi, and animals [20]. These photosynthetic pigments consist of two classes of molecules: carotenes and xanthophylls. Carotenoids play an important role in photosynthetic light-harvesting complexes; they absorb the solar spectrum in the blue-green region and transfer the energy to chlorophylls [21]. Furthermore, carotenoids also act as a photoprotector in photosynthetic organisms. Many studies have reported a strong correlation between increased UVB irradiation and carotenoid accumulation in terrestrial and marine plants [22,23]. As an example, Hupel et al. (2011) demonstrated that UVB irradiation increased the carotenoid contents in brown algae *Pelvetia canaliculata.*

**Figure 2:**
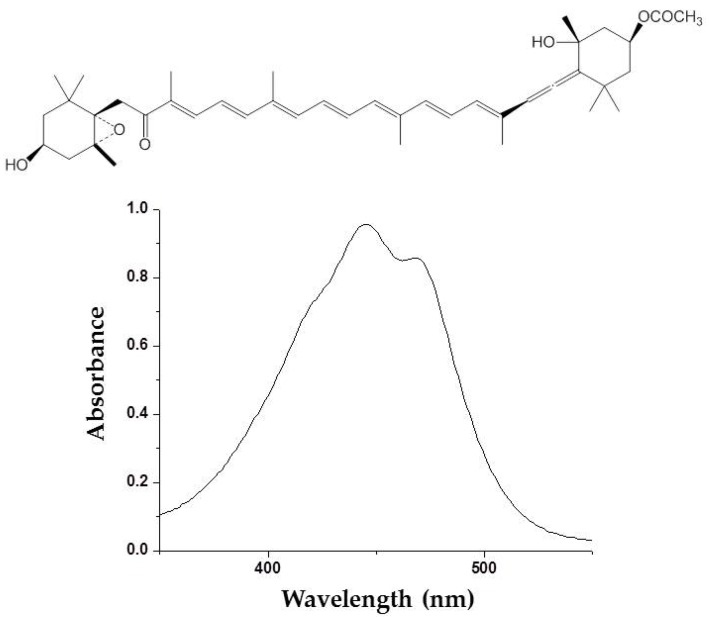
Molecular structure and ultraviolet (UV) absorption spectra of fucoxanthin from brown algae.

Photoprotective effects of fucoxanthin (Figure 2) derived from marine brown algae against UVB-induced photoaging have been reported [24]. Photoprotective activity of fucoxanthin has been determined by various in vitro and in vivo methods such as comet assay, human dermal fibroblast, and hairless mice irradiation. ROS scavenging activity is mainly considered to be a mechanism of action underlying the photoprotective activity of fucoxanthin [25,26,27]. Carotenoids, including fucoxanthin, are known as a singlet oxygen quencher. These photosynthetic pigments mitigate the harmful effects associated with UV irradiation by dissipating the excess energy as heat and returns to the initial ground state. Recently, fucoxanthin has been demonstrated to stimulate filaggrin promoter activity in UV-induced sunburn [28]. Filaggrin is a UV-sensitive gene that reflects the state of the skin damage. This stimulation of a UV-sensitive gen promotor by fucoxanthin suggested that other protective mechanisms of fucoxanthin might be exerted by the promotion of skin barrier formation through the induction of UV-sensitive gene expression.

Photoprotection mechanisms of fucoxanthin might also be achieved by oral administration. It has been shown that photodamage on the skin or eyes can be protected by biological compounds in tissues, which come from nutritional sources via the bloodstream. Stahl and Sies (2012) reported the concentration of carotenoids in human skin and demonstrated that there are considerable differences in the patterns in each skin layer. As an example, high concentrations of carotenoids are found in the skin of the forehead, the palm of the hand, and dorsal skin. Meanwhile, lower concentrations are found in the skin of the arm and the back of the hand of the human body [29]. In the human body, fucoxanthin absorption strongly depends on a number of factors, including the amount and type of dietary lipids consumed, the stability of the matrix to which the carotenoid is bound, and additional dietary factors such as dietary fiber. The esterified fucoxanthin is likely to be incorporated into the lipid core in chylomicron and carried into a variety of tissues, including the skin [30]. 

Recently, it has been reported that skimmed milk is an excellent food matrix for fucoxanthin application in terms of stability and bioavailability [31]. An in vivo pharmacokinetic study with a single oral administration of fucoxanrhin fortified in skimmed milk showed the highest absorption of fucoxanthinol and amarouciaxanthin A (two prime metabolites of fucoxanthin). Considering the potency of fucoxanthin as a photoprotective substance, further research studies are needed in order to verify photoprotective mechanisms of fucoxanthin oral consumption and the bioavailability of fucoxanthin (and its derivatives) in human skin.

### 2.3. Mycosporine Like Amino Acids

Mycosporine-like amino acids (MAAs) are low-molecular-weight, water-soluble molecules with maximum absorption bands in the UV spectrum between 310 and 360 nm. These molecules can be found in cyanobacteria, phytoplankton, lichens, gorgonians, cnidarians, sponges, shrimp, sea urchins, starfish, clams, ascidians, and marine algae. Most of the MAA-producing marine algae are red algae, followed by brown and green algae, respectively [32]. The type and accumulation of MAAs in marine algae varied based on season, climate, depth, and environmental variables (i.e., salinity, temperature, and nutrient availability) [33]. Unlike photosynthetic pigments, MAAs were invoked to function as passive shielding substances by dissipating the absorbed radiation energy in the form of harmless heat without generating photochemical reactions. In the organisms, MAAs not only function as “nature’s sunscreen compounds” but also serve as antioxidant molecules scavenging toxic oxygen radicals [34]. Up to now, more than 30 different chemical structures of MAAs have been elucidated. Table 1 present major MAAs identified from marine red algae.

MAAs have been reported as the strongest UVA-absorbing compounds in nature [42]. These low-molecular-weight molecules have gained considerable attention as highly active photoprotective candidates. Among other MAAs, porphyra-334 has been extensively studied. Daniel et al. (2004) reported that cream with 0.005% MAAs containing porphyra-334 can neutralize photodamage of UVA as efficiently as a cream with 1% synthetic UVA filters and 4% UVB filters [42]. In addition, porphyra-334 has been demonstrated to suppress ROS formation and downregulate the expression of MMP-1 and -13 on human dermal fibroblast following UVA irradiation. No adverse side effects have been reported from the treatment of porphyra-334 at concentration ≤200 μM on human skin fibroblasts. The formulation of porphyra-334 has been reported to increase photoprotective activity of sunscreen formula [41]. MAAs protect the skin cell due to their ability to disperse the harmful UV into heat that dissipates into the surroundings without forming reactive photoproducts. In addition, MAAs have also been reported as strong antioxidant molecules [40]. Hence, MAAs derived from marine algae can be recommended as photoprotective materials for skin care products.

### 2.4. Polyphenolic Compounds

**Figure 3:**
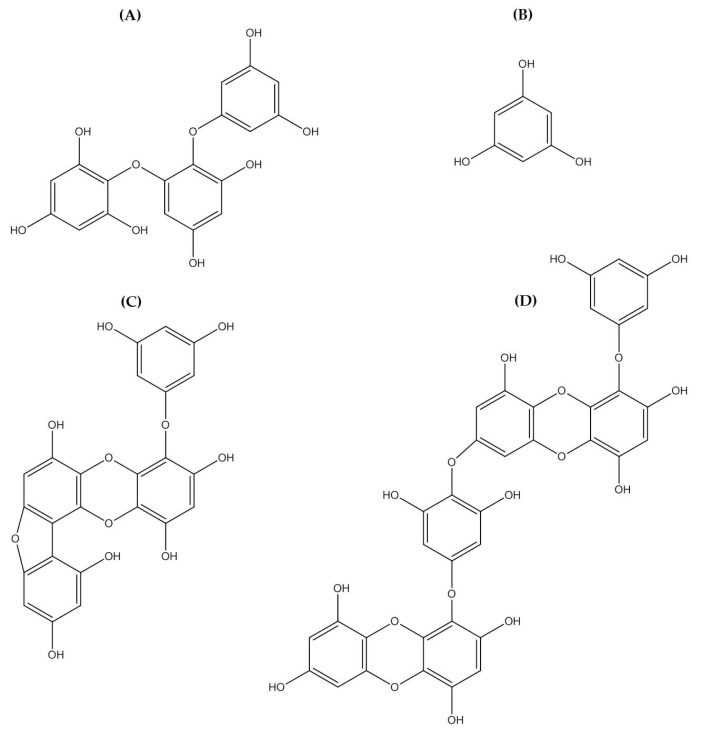
Chemical structure photoprotective polyphenol isolated from marine brown algae. Triphlorethol-A (**A**), phloroglucinol (**B**), fucofuroeckol-A (**C**), and dieckol (**D**).

**Figure 4:**
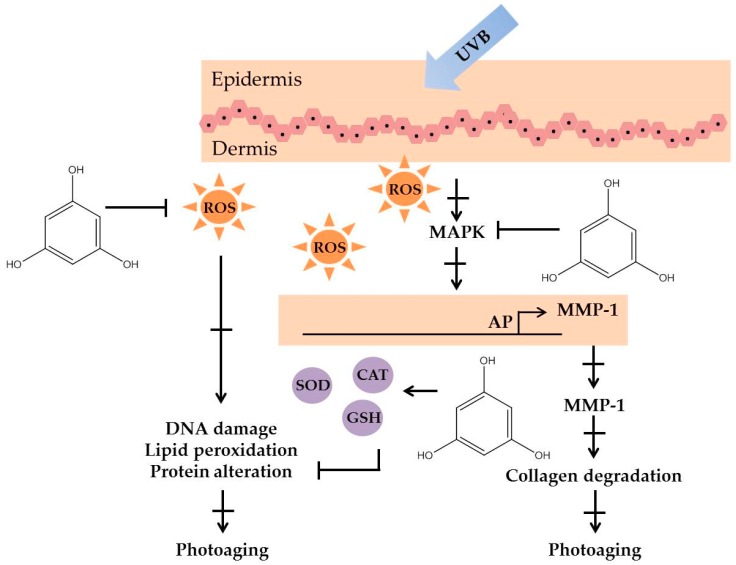
The photoprotective mechanism of phloroglucinol derived from marine algae.

Polyphenolic compounds are a class of secondary metabolites with diverse biological functions. These bioactive substances are divided into several classes according to the number of phenol rings and structural elements that bind these rings to one another [43]. The three main groups of polyphenols are phenolic acids, flavonoids, and tannins. Marine algae-derived polyphenols have been investigated for their photoprotective activities. Dieckol, phloroglucinol, fucofuroeckol-A, and triphlorethol-A (Figure 3) isolated from marine brown algae exhibited prominent protective effect against photodamage induced by UVB radiation, as demonstrated in many studies [44,45,46,47,48]. In order to understand the cellular and molecular photoprotective mechanisms of phloroglucinol, Piao and his colleagues developed it in UVB-irradiated mice and a HaCaT cell model. Phloroglucinol (10 μM) scavenged free radical and protects macromolecules damage in UVB-irradiated HaCaT cells [49]. In addition, phloroglucinol treatment significantly inhibited the UVB-induced upregulation of MMP-1 and phosphorylation of mitogen-activated protein kinases (MAPK) and activator protein-1 (AP-1) binding to the MMP-1 promoter [50]. Phloroglucinol has been demonstrated to be safe and effective when applied in the mouse skin irradiated with UVB [51]. Photoprotective activity of phloroglucinol is shown in Figure 4. The findings confirm the effectiveness of phloroglucinol as potential cosmeceutical leads for the formulations of sun-protective lotions and creams.

Polyphenols are bioactive substances characterized by the presence of more than one phenolic group (a hydroxyl group bound to an aromatic ring). Based on several reports, we assumed that their photoprotection activity is strongly correlated with their radical scavenging activity. The hydroxyl (–OH) group bound to the aromatic ring acts as an electron donor, giving it to a free radical or other reactive species. This underlies the inhibition of ROS and ROS-mediated damage on macromolecules, which in turn inhibit the activation of the signal transduction pathways such as the MAPK signaling pathway.

As mentioned in the many scientific reports, polyphenolic compounds represent an interesting class of active substance in the protection of UV-light-induced skin damage. Up to a certain concentration, marine algal-polyphenol did not exert any toxic effect, anticipating its potential use as a safe photoprotector that can be utilized in skin care products.

### 2.5. Marine Algae Extracts and Fractions

Extraction of active components from plant materials is the first and most important step in the development of photoprotective substances. Marine algae have been extracted with various solvents and investigated for their photoprotective effects (Table 2). Guinea et al. (2012) investigated the photoprotective potential of 21 commercial marine red and brown algae originated from Chile, Spain, South Africa, Argentina, Ireland, and Tonga. Compared to other extracts, two marine red algae *Macrocystis pyrifera* and *Porphyra columbina* exhibited the highest photoprotective activity [52]. Many studies have reported that certain species of marine algae can protect the skin against UVB-induced photoaging and damage due to antioxidant properties and their UV absorbing capacity. In addition, the photoprotection of marine algae extract has been correlated with MAAs and polyphenol constituents. As an example, *Porphyra yezoensis* extract showed photoprotective activity on the UVB-exposed HaCaT cells and human keratinocytes. The *Porphyra yezoensis* extract showed absorbance spectrum characteristics of MAAs in red algae and contained high phenolic compounds [53]. Polyphenolic compounds are generally more soluble in polar organic solvents, so organic solvents such as ethanol and methanol can be considered as effective extractants of polyphenolic components from marine algae. Supporting this hypothesis, aqueous extract of marine green algae (*Halimeda incrassate*) and red algae (*Bryothamnion triquetrum*) showed no photoprotective activity in UVC-irradiated plasmids [54]. 

Synthetic UV filters are used in skin care products to prevent photodamage and skin cancer. However, UV filters still have to be complemented by other compounds to make sun protection skin care more efficient to photodamage and skin photoaging. The combination of *Porphyra umbilicalis* extracts and *Ginkgo biloba* has been demonstrated to improve the photoprotective performance of sunscreens, which then prevent UV-induced photodamage [55]. Thus, marine algae can be considered potent materials for an effective photoprotective formulation with anti-aging properties. Photoprotective activity of marine red and brown algae have been characterized in many studies; however, up to now very little attention has been given to unraveling photoprotective substances from marine green algae. 

## 3. The Development of Photoprotective Compounds-Derived from Marine Algae

Organic solvent extraction is the most common technique to isolate photoprotective substances from marine algae. Extraction conditions, such as temperature, sample-to-solvent ratios, and extraction time, must then be adjusted in order to optimize the extraction process. Organic solvent such as ethanol, methanol, acetone, and ethyl acetate can be used for the extraction of photoprotective substances [15]. However, in the last few decades, the volume of solvents used in the chemical process is extremely concerning. Organic solvents are a major contributor to the overall toxicity potential associated with many industrial processes and to the waste generation of chemical industries. The disposal of excessive solvent to the environment significantly contributes to the release of greenhouse gases and other emissions [66]. Both academic and industrial researchers have therefore focused on minimizing solvent consumption through the development of solvent-free processes. Environmentally friendly “blue biotechnologies” such as EAE, UAE, MAE, SC–CO_2_, and SWE have been demonstrated as potential technologies to obtain photoprotective compounds from marine algae. Table 3 shows advantages and disadvantages of blue biotechnologies to obtain photoprotective substances from marine algae. 

### 3.1. Enzyme-Assisted Extraction

The EAE technique has been widely used to improve the extraction efficiency of bioactive substances from terrestrial plants. On the contrary, the application of the EAE method to extract photoprotective substances from marine algae has rarely been reported. The EAE allows preparation of bioactive substances from marine green, brown, and red algae [67]; however, physico-chemical conditions of the reaction media, such as temperature, pH of the protein solution, and enzyme ratios, must then be adjusted in order to optimize the activity of the enzyme. Proteolytic enzymes from different sources such as microbes, plants, and animals can be used for the hydrolysis process of marine algae [68]. Crude polysaccharide from the brown algae *Ecklonia cava* has been recovered by using EAE. Lyophilized *E. cava* was ground and sieved to obtain a smaller particle size, and this produced higher extraction yields. Hence, in addition to physic-chemical conditions of reaction media, surface areas of the sample is another important factor in the EAE process [69]. Recently, it was reported that the EAE process increases antioxidant activity of fucoidan from marine brown algae *Cystoseira trinodis* [70]. Alternative extraction conditions such as EAE can be successfully employed in order to degrade marine algae tissues on the basis of the recovery of bioactive compounds with a considerably high yield. 

### 3.2. Ultrasound Assisted Extraction and Microwave Assisted Extraction

The established extraction technology that can be used to isolate photoprotective substances from marine algae is MAE and UAE. Both are energy input-assisted extraction methods and have been used to isolate bioactive substance from terrestrial plant material for many years [71]. MAE involves the heating process of a solution in contact with a sample using microwave energy. Different from classical heating, microwaves heat the sample simultaneously without heating the vessel. Therefore, the solution reaches its boiling point very rapidly, leading to very short extraction times [72]. The high recovery of fucoidans derived from *Ascophyllum nodosum* by MAE has been reported [73]. The MAE of fucoidan from *Ascophyllum nodosum* at 90 °C has a similar composition, molecular weight, and reducing power than native fucoidan extracted by the conventional method. It is assumed that the sulfate contents were only affected to the extraction temperature of fucoidan. In addition to MAE, UAE has also been reported to improve the yield of fucoidan with antioxidant activities from marine brown algae, *Sargassum muticum* [74]. The UAE principle is based on the waves migrating through a medium inducing pressure variations. Notably, considering that the energy input of MAE and UAE can exceed the energy level required for the cleavage of the sulfate esters, it is recommended that the necessary energy input be temporarily exerted during the extraction process to avoid any structural alterations to the sulfated polysaccharide [72].

### 3.3. Supercritical Carbon Dioxide

**Figure 5:**
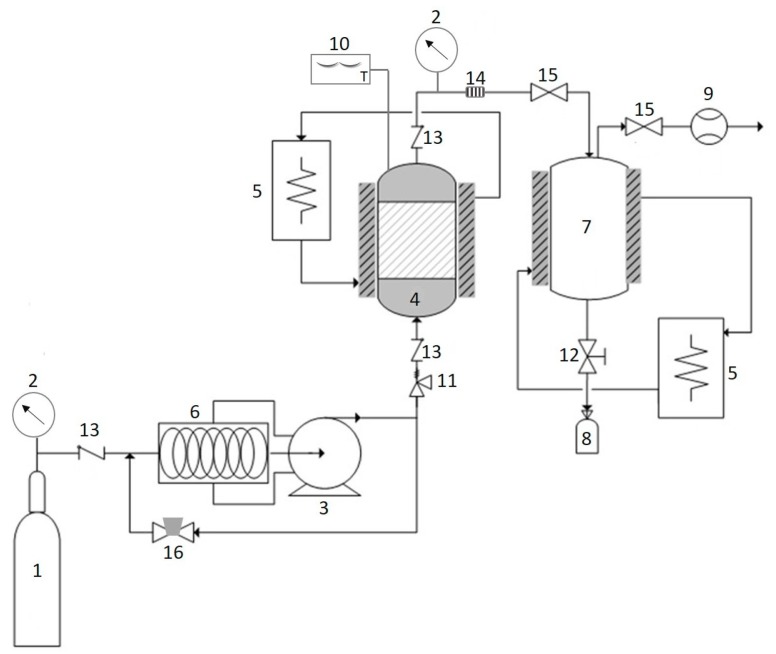
Schematic diagram of SC–CO_2_. The CO_2_ tank (1); pressure gauge (2); high pressure pump (3); extractor (4); heat exchanger (5); chiller (6); separator (7); sample collector (8); flow meter (9); digital thermometer (10); safety valve (11); needle valve (12); check valve (13); filter (14); metering valve (15); back pressure regulator (16).

Recently, scientists and industrialists have paid a great deal of attention to the application of SC–CO_2_ fluid (Figure 5), a hydrophobic and environmentally friendly medium, as an alternative to conventional organic solvent extraction [75]. SC–CO_2_ is a promising method for the recovery of photoprotective substances from marine algae, which can be carried out under mild operating conditions. The SC–CO_2_ process offers new opportunities for the solution of separation problems as it is a nontoxic, nonflammable, inexpensive, and clean solvent [76]. The higher carotenoids yield from *Saccharina japonica* and *Sargassum horneri* obtained by SC–CO_2_ as compared to the conventional extraction has been reported [77]. The solvating capacity of SC–CO_2_ fluid can be controlled by manipulating pressure and temperature to give suitable selectivity. Therefore, the temperature and pressure applied greatly affected the carotenoids solvating power of SC–CO_2_ and hence the yield of carotenoids. Co-solvent has also been demonstrated to increase the yield of bioactive substances from marine algae [78,79]. As an example, Roh et al. (2008) and Conde et al. (2014) reported that the use of ethanol as co-solvent in the SC–CO_2_ process increases phenolic and fucoxanthin yield from marine brown algae, *Undaria pinnatifida*, and *Sargassum muticum*, respectively. More recently, sunflower oil has been shown to improve the extraction yield of carotenoids and fucoxanthin from *Saccharina japonica* [80]. 

### 3.4. Subcritical Water Extraction

The SWE process is an environmentally clean technique that can be used to recover photoprotective substances from marine algae. During SWE, water is maintained in the subcritical state, between its boiling point (100 °C and 0.10 MPa) and critical point (374 °C and 22 MPa), where it remains as a liquid due to the high pressure [81]. High yield of fucoidan and carrageenan extracted by SWE has been demonstrated [82,83]. Generally, the SWE process increases the yield of bioactive substances and their biological activities. However, lower physical properties such as gelling and viscosity were also observed; these might be due to the degradation of polysaccharidea in higher temperature. The SWE process can be used, in addition to recovering photoprotective substances, to modify the structure of those substances. Meillisa et al. (2015) demonstrated structure modification of alginate from *Saccharina japonica* by SWE. Considering the blue extraction technique, SCE exhibits a number of advantages over conventional organic solvent extraction. The important advantages of this method include its simplicity, reduced extraction time, lower cost of the extracting agent, and its environmental friendliness. 

## 4. Conclusions and Future Prospects of Photoprotective Substances from Marine Algae

Marine algae are the subject of increasing interest for their potential as a source of bioactive substances in cosmetics industries for several reasons. First, these marine organisms are considered as the fastest growing organisms on Earth. Extensively available marine areas are potential areas for marine algae aquaculture. Moreover, marine algae aquaculture techniques of commercial species (i.e., *Eucheuma cottonii*, *Laminaria japonica*, *Ecklonia cava*, *Gracilaria* sp.) have developed rapidly in the last decades. Marine algae can be found from tropical, cold-temperate areas to polar areas. This great number of biodiversity can be seen as a potential field for the blue exploration of marine algae. In addition, marine algae have exhibited unique chemical structures unlike those found in terrestrial counterparts. These organisms are viewed as “natural and healthy” by many people, and this promotes a positive response for consumers, who often regard natural and nontoxic entities. Many species of marine algae have been used as extracts in cosmetics, and there is no restriction for cosmetic use. Hence, marine algae may be considered a consumer-friendly source of skin care and cosmetic products that may be used for photoprotection. However, the health claims of photoprotective substances derived from marine algae that have been reported by many studies are mostly acquired only through in vitro and in vivo studies, so comprehensive studies on the mode of photoprotective action, biological consequences, and possible side effects have to be conducted in order to use those functional materials as skin care and cosmetic products. Photoprotective activity of orally administered bioactive substances from marine algae has also been reported. These findings reveal the potential of the development of photoprotective supplements and/or pharmaceuticals derived from marine algae. 

The use of blue biotechnologies to recover photoprotective substances is becoming very important since the volume of solvents used in the chemical process is extremely concerning. Further, advances in molecular biology and aquaculture technologies such as Integrated Multi Trophic Aquaculture (IMTA) and Recirculating Aquaculture systems (RAS) are important to bridge the gap between the challenges pertaining to the exploitation of marine algae. Particularly, the aquaculture techniques of many brown algae species still remains a challenge and are necessary for the sustainable use of marine algal metabolites and for reduced production cost. Collectively, we predict that extensive application of marine algae in skin care, cosmetics, and pharmaceuticals with advanced photoprotective benefits is not a distant prospect.

## Figures and Tables

**Table 1:** Mycosporines-Like amino acids (MAAs) identified in marine algae.

Mycosporine-like Amino Acids	Marine Algae	Reference
Shinorine 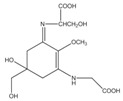	*Gloiopeltis fucatas; Mazzaella* sp.; *Gracilaria vermiculophylla; Palmaria palmata; Porphyra* sp.; *Porphyra umbilicalis*	[35,36,37]
Palythine 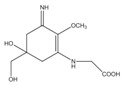	*Gloiopeltis fucatas; Mazzaella* sp.; *Gracilaria vermiculophylla; Palmaria palmata; Porphyra* sp.; *Porphyra umbilicalis*	[35,36,37,38]
Porphyra-334 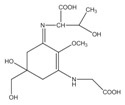	*Gracilaria vermiculophylla; Palmaria palmata; Porphyra* sp.; *Porphyra umbilicalis; Poprphyra yezoensis; Porphyra vietnamensis*	[35,37,38,39,40,41]
Asterina-330 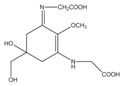	*Gracilaria vermiculophylla*	[35,38]
Mycosporine-glycine 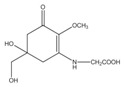	*Mazzaella laminarioides*	[38]

**Table 2:** Summary of photoprotective effects of marine algae extracts.

Class	Species	Origin	Extract/Fraction	Activity	Reference
Red algae	*Solieria chordalis*	France	MeOH extract/CPC fractionation *n*-heptane/EtOAc//MeOH/dW (19/1//19/1; *v*/*v*)	UVB absorption & free radical scavenging activity	[56]
	*Porphyra umbilicalis*	France	Cosmetic formula (5% extract)	Protect UV-radiated skin from erythema	[55]
	*Porphyra yezoensis*	Korea	EtOH extract (80%)/chloroform/MeOH/dW (2/1/0.9)	Modulate viability of UVB-exposed HaCaT	[53]
	*Gelidium amansii*	Korea	MeOH extract and fermentation	Protect skin photoaging in Hairless Mice induced by UVB	[57]
	*Polyopes affinis*	Korea	EtOH extract	Inhibit UVB-induced ROS in HaCaT	[58]
	*Solieria chordalis*	France	EtOAc extract	Protect synthetic chlorophyll solution from UVB	[59]
	*Polysiphonia morrowii*	Korea	EtOH extract (80%)	Protect HaCaT from UVB-induced cell damage	[60]
	*Chondracanthus tenellus*	Korea	EtOH extract (80%)	Protect HaCaT from UVB-induced cell damage	[61]
	*Bonnemaisonia hamifera*	Korea	EtOH extract (80%)	Protect HaCaT from UVB-induced cell damage and inhibit ROS	[62]
	*Lomentaria hakodatensis*	Korea	EtOH extract (80%)	Protect HaCaT from UVB-induced cell damage	[63]
	*Macrocystis pyrifera*	Argentina	Ace extract	UVB protection on zebrafish embryo	[52]
	*Porphyra columbina*	Argentina	Ace extract	UVB protection on zebrafish embryo	[52]
Brown algae	*Sargassum muticum*	Korea	EtOAc fraction	Inhibits wrinkle formation in UVB-induced mice (in vivo)	[64]
	*Sargassum muticum*	Korea	EtOAc fraction	UVB irradiated human keratinocytes (in vitro)	[65]
	*Undaria crenata*	Korea	EtOH extract (80%)	Protect HaCaT from UVB-induced cell damage	[15]
	*Lessonia vadosa*	Argentina	Ace extract	UVB protection on zebrafish embryo	[52]
	*Lessonia nigrescens*	Chile	Ace extract	UVB protection on zebrafish embryo	[52]
	*Ecklonia maxima*	South Africa	Ace extract	UVB protection on zebrafish embryo	[52]
	*Durvillaea antarctica*	Chile	Ace extract	UVB protection on zebrafish embryo	[52]
	*Fucus vesiculosus*	Spain	Ace extract	UVB protection on zebrafish embryo	[52]
	*Saccharina latissima*	Spain	Ace extract	UVB protection on zebrafish embryo	[52]
	*Ascophyllum nodosum*	Ireland	Ace extract	UVB protection on zebrafish embryo	[52]

**Table 3:** Technologies for the recovery of photoprotective substances from marine algae.

Techniques	Advantage	Disadvantage	Target Photoprotective Substances
Organic solvent	Easy to operate	Environmental waste Cost of organic solvent Clean up step needed	Carotenoids, Phenolics, MAAs, Sulfated Polysaccharides, Extracts
EAE	No harmful solvents High yield Mild process	Extracted substances required further process Cost of the enzymes Optimization of enzymatic process Clean up step needed	Extracts, Sulfated Polysaccharides
UAE & MAE	Reduce extraction time Low solvent	High power consumption Scaling up is difficult Clean up step needed	Sulfated Polysaccharides
SC–CO_2_	Reduce extraction time Simple process Environmental friendly Low operating temperatures (40–60 °C) Clean final product Low solvent	Cost of the installations Required special manpower Optimization process	Carotenoids (i.e., fucoxanthin);
SWE	Reduce extraction time Simple process Environmental friendly High yield Low solvent	Cost of the installations Clean up step needed Elevated temperatures	Sulfated polysaccharides (i.e., carrageenan; fucoidan); polyphenols
